# Occult hepatitis B virus infection in patients with chronic liver disease of different etiology in a Brazilian referral center: comparison of two different hepatitis B virus deoxyribonucleic acid amplification protocols: a cross-sectional study

**DOI:** 10.1590/1516-3180.2022.0147.R1.12072022

**Published:** 2022-09-23

**Authors:** Alessandra Coutinho de Faria, Bernardo Henrique Mendes Correa, Luciana Costa Faria, Paula Vieira Teixeira Vidigal, Marcelo Antônio Pascoal Xavier, Teresa Cristina Abreu Ferrari

**Affiliations:** I MD, MSc. Physician, Department of Internal Medicine, Faculty of Medicine, Hospital das Clínicas, Universidade Federal de Minas Gerais (UFMG), Belo Horizonte (MG), Brazil.; II Research Associate, Undergraduate Student, Department of Internal Medicine, Faculty of Medicine, Universidade Federal de Minas Gerais (UFMG), Belo Horizonte (MG), Brazil.; III MD, PhD. Professor Associate, Department of Internal Medicine, Faculty of Medicine, Universidade Federal de Minas Gerais (UFMG), Belo Horizonte (MG), Brazil.; IV MD, PhD. Professor Associate, Department of Pathological Anatomy and Forensic Medicine, Faculty of Medicine, Universidade Federal de Minas Gerais (UFMG), Belo Horizonte (MG), Brazil.; V MD, PhD. Professor, Department of Pathological Anatomy and Forensic Medicine, Faculty of Medicine, Universidade Federal de Minas Gerais (UFMG), Belo Horizonte (MG), Brazil.; VI MD, PhD. Professor, Department of Internal Medicine, Faculty of Medicine, Universidade Federal de Minas Gerais (UFMG), Belo Horizonte (MG), Brazil.

**Keywords:** Hepatitis B virus, Liver diseases, Polymerase chain reaction, Occult hepatitis B infection, HBV DNA, Chronic liver disease, Nested-PCR

## Abstract

**BACKGROUND:**

Occult hepatitis B virus infection (OBI) is defined as the presence of hepatitis B virus (HBV) deoxyribonucleic acid (DNA) in the liver of individuals with undetectable hepatitis B virus surface antigen (HBsAg) in the serum. The actual prevalence of OBI and its clinical relevance are not yet fully understood.

**OBJECTIVE:**

To evaluate the prevalence of HBV DNA in liver biopsies of HBsAg-negative patients with chronic liver disease of different etiologies in a referral center in Brazil and compare two different HBV DNA amplification protocols to detect HBV.

**DESIGN AND SETTING:**

This cross-sectional observational study was conducted at the Liver Outpatient Clinic, Hospital das Clínicas, Universidade Federal de Minas Gerais, Belo Horizonte, MG, Brazil, between January 2016 and December 2019.

**METHODS:**

HBV DNA was investigated in 104 liver biopsy samples from individuals with chronic liver disease of different etiologies, in whom HBsAg was undetectable in serum by nested-polymerase chain reaction (nested-PCR), using two different protocols.

**RESULTS:**

OBI, diagnosed by detecting HBV DNA using both protocols, was detected in 6.7% of the 104 individuals investigated. Both protocols showed a good reliability.

**CONCLUSION:**

In addition to the differences in the prevalence of HBV infection in different regions, variations in the polymerase chain reaction technique used for HBV DNA amplification may be responsible for the large variations in the prevalence of OBI identified in different studies. There is a need for better standardization of the diagnostic methods used to diagnose this entity.

## INTRODUCTION

Hepatitis B virus (HBV) infection is one of the most prevalent infections worldwide and is an important cause of morbidity and mortality. It often progresses to chronic hepatitis, liver cirrhosis, and hepatocellular carcinoma (HCC) and is responsible for approximately 780,000 deaths annually.^1–4^

HBV infection is usually diagnosed based on the presence of the HBV surface antigen (HBsAg) in the serum. However, the possibility of persistence of the HBV genome in HBsAg-negative individuals has been demonstrated. This entity termed occult hepatitis B virus infection (OBI), is defined by the presence of HBV deoxyribonucleic acid (DNA) in the liver (in some cases, also in the serum) in the absence of circulating HBsAg.^1,5,6^ When HBV DNA is detectable in the serum, its levels are usually very low (< 200 IU/mL). It has been hypothesized that OBI is related to strong suppression of viral activity by host immune surveillance.

From a biomolecular perspective, different mechanisms may be involved in OBI development: mutations in the HBsAg gene, epigenetic changes, host immune responses, human immunodeficiency virus (HIV) and hepatitis C virus (HCV) coinfections, metabolic factors, HBV immune complexes, and genomic integration.^7–12^ Moreover, there is evidence that microRNAs (miRNAs) are differentially expressed in patients with OBI compared to healthy controls.^13^

The exact magnitude, pathogenesis, and clinical relevance of OBI are not completely understood. Individuals with this entity can transmit HBV through blood transfusion or organ transplantation.^1,5,6,11,14,15^ In the setting of immunosuppression, the suppressed state of viral activity observed in OBI can be discontinued, leading to the development of typical hepatitis B, which often has a severe course.^16,17^ Observational data suggest that OBI may favor or accelerate the progression of other chronic liver diseases, such as HCV infection,^18^ and HCC development.^1,6,19,20^

The diagnosis of OBI has been established using polymerase chain reaction (PCR) to amplify HBV DNA. Modifications to the PCR technique (nested-PCR and real-time PCR) were used to increase the sensitivity of the method. PCR assays vary in sensitivity and specificity, and the factors associated with the biological material in which the DNA is probed may affect HBV detection rate. Thus, the diagnosis of OBI remains challenging because there is no standard method or protocol for the detection of occult HBV DNA.^21^

In Brazil, few studies have evaluated the prevalence of OBI using current case definition criteria.^24–27^

## OBJECTIVE

In this context, the present study aimed to investigate the frequency of OBI in patients with chronic liver disease who underwent liver biopsy as part of the investigation of their disease and to compare two different HBV DNA amplification protocols for HBV detection.

## METHODS

This is a cross-sectional observational study approved by the Research Ethics Committee of the Universidade Federal de Minas Gerais (UFMG) (CAAE 32140914.0.0000.5149) on October 1, 2014. All the patients signed an informed consent form.

### Patients

Liver biopsy samples were selected from 104 adult patients, HBsAg-negative, with chronic liver disease of any etiology, who had undergone liver biopsy as part of the investigation of their disease and were followed up at the Liver Outpatient Clinic, Hospital das Clínicas, UFMG, between January 2016 and December 2019.

In addition to the paraffin-embedded biological samples, data from medical records were collected, including the results of markers of previous HBV infection, collected at the time of biopsy, that is, antibodies anti-HBV core antigen (anti-HBc) and antibodies anti-HBV surface antigen (anti-HBs), this last marker from unvaccinated patients. The exclusion criteria were HIV infection, use of immunosuppressive drugs, and hematological malignancies.

Patients were grouped according to the etiology of the underlying liver disease as follows: chronic liver disease associated with HCV, nonalcoholic steatohepatitis, autoimmune liver disease (autoimmune hepatitis, primary biliary cholangitis, and primary sclerosing cholangitis), cryptogenic liver disease and hemochromatosis.

### Representativeness of liver biopsies

The representativeness of liver biopsies was assessed based on fragment size and number of portal tracts. The size distribution of the fragments was very close to a normal curve, with a mean size of approximately 13 mm.

**Figure 1:**
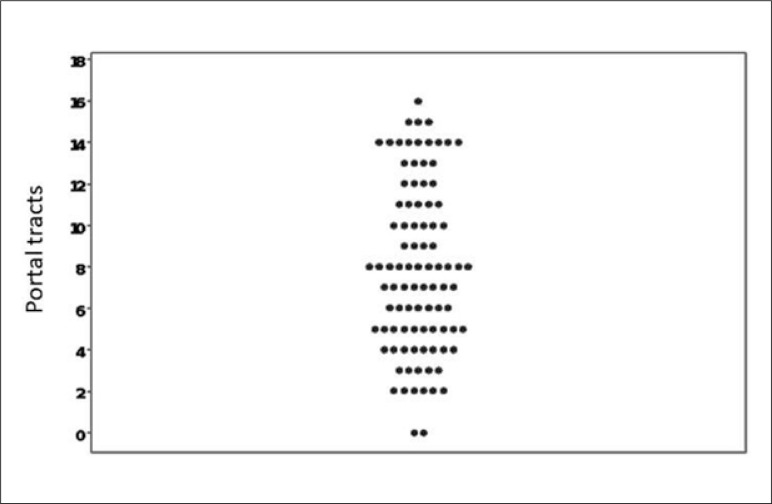
Number of portal tracts.

The distribution of the number of portal tracts, unlike the biopsy size, showed wide variability with a skewed distribution, despite the higher concentration around the eight portal tracts (Figure 1).

**Figure 2:**
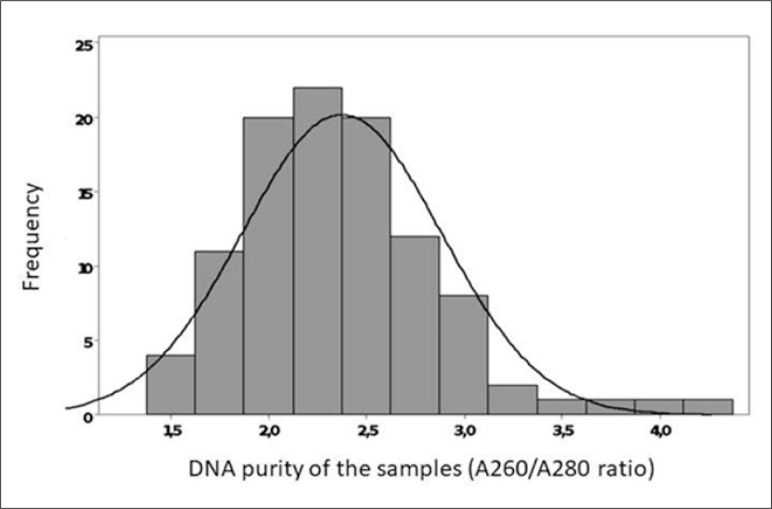
Deoxyribonucleic acid (DNA) purity in the samples (A260/A280 ratio).

The quality of the DNA present in the samples was analyzed using the A260/A280 ratio. Nucleic acids absorb light with a wavelength of 260 nm. Proteins absorb light with a wavelength of 280 nm. Thus, the A260/A280 ratio provides a parameter for evaluating the quality of nucleic acid preparation. DNA was considered pure when the A260/A280 ratio was between 1.8 and 2. Values lower than 1.8 indicate protein contamination. Figure 2 shows that the DNA was not of good quality.

### DNA extraction and amplification

DNA was extracted from paraffin-embedded samples using the Qiamp DNA FFPE Tissue Kit (QIAGEN, Hilden, Germany), as recommended by the manufacturer. DNA was extracted from three negative and positive controls. DNA from all samples was amplified according to two previously published protocols: the protocols described by Raimondo et al.^5^ (protocol 1) and Chapel et al.^28^ (protocol 2).

According to protocol 1, DNA was amplified using nested PCR and the primers employed were complementary to four conserved regions of the viral genome (pre-S/S, pre-core/core, polymerase, and region X), as described in Table 1. A programmable thermal controller PTC-100™ thermal cycler (MJ Research, Inc., St. Bruno, Canada) was used for PCR.

**Table 1:** Initiators for hepatitis B virus deoxyribonucleic acid detection by nested-polymerase chain reaction - protocol 1^5^

Hepatitis B virus genomic regions		Nucleotide positions
**S region**		
	S1- F: 5′-CATCAGGATTCCTAGGACCCCT-3′	[168–189]
	S2- F: 5′-CTTGTTGACAAGAATCCTCACA-3′	[214–235]
	S3-R: 5′- AGGACAAACGGGCAACATAC-3′	[478–458]
	S4-R:5′- CCAACAAGAAGATGAGGCATA-3′	[442–420]
**C region**		
	C5-F: 5′- TCACCTCTGCCTAATCATC-3′	[1825–1843]
	C6-F: 5′- TTCAAGCCTCCAAGCTGTGCC-3′	[1862–1882]
	C7-R: 5′- GAGGGAGTTCTTCTTCTAGG-3′	[2391–2371]
	C8-R: 5′- AGGAGTGCGAATCCACACTCC-3′	[2277–2267]
**Polymerase region (Pol)**		
	P9-F: 5′- CGTCGCAGAAGATCTCAATC-3′	[2420–2439]
	P10-F: 5′- CCTTGGACTCATAAGGT-3′	[2463–2479]
	P11-R:5′- TCTTGTTCCCAAGAATATGGT-3′	[2845–2825]
	P12-R: 5′- TCCCAAGAATATGGTGACCC-3′	[2839–2820]
**X region**		
	X13-F: 5′- CGCCAACTTACAAGGCCTTTC-3′	[1100–1120]
	X14-F: 5′- CCATACTGCGGAACTCCTAG-3′	[1266–1685]
	X15-R: 5′-GGCGTTCACGGTGGTCTCCAT-3′	[1628–1608]
	X16-R: 5′- CGTAAAGAGAGGTGCGCCCC-3′	[1540–1521]

For internal and external reactions, a SuperMix Kit (PCR SuperMix, Invitrogen, Fisher Scientific, Inc., Fair Lawn, United States) was used. The reaction conditions were as follows: initial denaturation at 94 °C for two minutes, followed by 35 cycles of denaturation at 94 °C for 30 s, primer binding at 56 °C for 45 s, extension at 72 °C for 90 s, and a final step of 10 min at 72 °C.

The primers used in protocol 2 are listed in Table 2. DNA was amplified by nested PCR, using the same kit for internal and external reactions. Primers for the S and Pol regions of the viral genome were used in the following order: in the first amplification step, primers 14/13 were used to amplify a 416 bp sequence located in a conserved region of the polymerase (Pol) and surface (S) genes. For the second step, the primers 06/03 were used to re-amplify a 128 bp segment located in the 416 bp sequence. The external reaction parameters (primers 14/13) were as follows: initial denaturation at 90 °C for seven min, 40 cycles of 20 s at 94 °C, 60 s at 47 °C, 60 s at 74 °C; and a final extension at 74 °C for seven min. Five microliters of the products from the first reaction were subjected to 35 cycles of a second PCR reaction using the primers 06/03; the parameters of this second step (internal reaction) were: 20 s at 94 °C, 60 s at 57 °C, and 60 s at 74 °C for each cycle, with an initial denaturation at 90 °C and a final extension step at 72 °C.

**Table 2:** Initiators for hepatitis B virus deoxyribonucleic acid detection by nested-polymerase chain reaction - protocol 2^28^

Primer sequences		Oligonucleotides position
**External reaction**		
	Primer 14″ F: 5′- ATCTTCTTATTGGTTCTTCT-3′	[430–449; pol]
	Primer 13″ R: 5′- GTTAGGGTTTAAATGTATAC-3′	[845–826; S]
**Internal reaction**		
	Primer 06 F: 5′-CTTGGATCCTATGGGAGTGG-3′	[632–651; pol]
	Primer 03 R: 5′-CTCAAGCTTCATCATCCATATA-3′	[759–738; S]

Polyacrylamide gel electrophoresis was performed to verify whether the fragment of interest was amplified by PCR.

### Statistical analysis

For this study, OBI cases were considered in individuals in whom DNA amplification was obtained using the two protocols.

Categorical variables are presented as numbers and percentages. Continuous variables were expressed as mean ± standard deviation, as they presented a normal distribution according to the Shapiro-Wilk test. Pearson's chi-square or Fisher's exact test was used to analyze the differences between qualitative data when appropriate. The Student's t-test was used to compare quantitative data. The degree of agreement between tests was calculated using the kappa coefficient of agreement. Statistical significance was set at P value < 0.05.

## RESULTS

### Epidemiological and clinical data

Of the 104 patients investigated, the mean age was 47.8 (range, 18–73). The patients' demographic, clinical, and laboratory characteristics are shown in Table 3.

**Table 3:** Characteristics of the 104 HBsAg-negative patients included in the study

Characteristic		Numerical value (n = 104)
**Gender (F/M)**		57 (54.8%)/47 (45.2%)
**Age (mean age in years** ± **SD)**		47.8 ± 12.6
**Blood transfusion** * **yes/no**		19 (23.5%)/62 (76.5%)
**Anti-HBc positive** ¶ **yes/no**		14 (16.7%)/70 (83.3%)
**Anti-HBs positive** § **yes/no**		37 (41.3%)/46 (58.7%)
**Fibrosis grade on liver biopsy** †		
	F0	42 (40.4%)
	F1	24 (23.1%)
	F2	16 (15.4%)
	F3	11 (10.6%)
	F4	11 (10.6%)

HBsAg = surface antigen of hepatitis B virus; F/M = female/male; SD = standard deviation, anti-HBc = antibody anti-hepatitis B virus core antigen; anti-HBs = antibody anti-hepatitis B virus surface antigen.

Data are presented as number (percentage) and mean ± standard deviation.

* Data available for 81 patients;

¶ data available for 84 patients;

§ data available for 83 patients.

† F0, no fibrosis; F1, portal fibrosis without septa; F2, few septa; F3, numerous septa without cirrhosis; F4, cirrhosis.^29^

The most common underlying liver disease was chronic hepatitis C (41.3%). No patient had HCC. In 84 cases, total anti-HBc data were available, of which 14 (16.7%) were positive. Anti-HBs were analyzed in 83 unvaccinated individuals and were positive in 37 (44.6%). Fourteen patients were positive for both markers, and 47 had negative markers.

### OBI diagnosed by nested-PCR

HBV DNA was amplified in 13 (12.5%) of the 104 patients evaluated using protocol 1 and in nine (8.7%) using protocol 2 (Table 4). Considering the cases identified by both protocols, the frequency of OBI was seven in 104 individuals (6.7%). In six cases, HBV DNA was amplified only by the Raimondo et al. protocol,^5^ and in two, only by the Chapel et al. one.^28^

**Table 4:** Amplification of HBV DNA according to two different protocols

HBV DNA	Protocol 1	Protocol 2
Positive	13 (12.5%)	9 (8.7%)
Negative	91 (87.5%)	95 (91.3%)
**Total**	**104 (100.0%)**	**104 (100.0%)**

HBV = hepatitis B virus; DNA = deoxyribonucleic acid.

Protocol 1: Raimondo et al., 2008;^5^ protocol 2: Chapel et al., 1995.^28^

Data are presented as numbers (percentage).

The value of the kappa coefficient of agreement, considering the comparison of protocols 1 and 2, was 0.595 (95% confidence interval [CI], 0.487–0.696), showing that there was a substantial agreement between the results obtained in both tests.

No difference was found in the mean age (P = 0.244) or sex distribution (P = 0.698) between patients with and without OBI. No association was found between OBI and any underlying liver disease (P = 0.169). Table 5 summarizes the distribution of OBI cases according to the etiology of underlying liver disease.

**Table 5:** Distribution of OBI cases according to the etiology of the underlying liver disease

Etiology	HBV DNA		Total
	Negative	Positive	
Chronic HCV infection	39 (90.7%)	4 (9.3%)	**43 (100.0%)**
NASH	21 (95.5%)	1 (4.5%)	**22 (100.0%)**
Autoimmune	26 (96.3%)	1 (3.7%)	**27 (100.0%)**
Cryptogenic	9 (90.0%)	1 (10.0%)	**10 (100.0%)**
Hemochromatose	2 (100.0%)	0 (0.0%)	**2 (100.0%)**
**Total**	**97 (93.3%)**	**7 (6.7%)**	**104 (100.0%)**

OBI = occult hepatitis B infection; HBV = hepatitis B virus; DNA = deoxyribonucleic acid; HCV = hepatitis C virus; NASH = nonalcoholic steatohepatitis.

Data are presented as numbers (percentage).

No association was observed between the occurrence of OBI and presence of anti-HBc antibodies (P = 0.086). However, such an association was observed when HBV DNA cases were identified using protocol 1. When comparing the patients with positive and negative PCR results (protocol 1) and the presence of HBV markers (anti-HBs and/or anti-HBc), it was observed that among the 13 individuals with positive HBV-PCR results, only one presented all negative markers, and 12 (92.3%) had at least one positive marker. Among 71 individuals with negative PCR results for HBV, 46 (64.8%) presented all negative markers and 25 (35.2%) presented with at least one positive antibody (anti-HBc and/or anti-HBs) (P = 0.000). No association was observed between the presence of HBV and hemotransfusion history (P = 1.000).

## DISCUSSION

OBI was detected in 6.7% of 104 individuals with HBsAg-negative chronic liver disease, considering only those cases in which HBV DNA was detected by both protocols. The presence of HBV in individuals with chronic liver disease varies from 0.7-73% in different countries.^18,28,30–34^ In Brazil, the range is 2%^35^ to 19.5%.^23^ This variation is probably due to differences in the prevalence of HBV infection in different regions of Brazil and the world and in the methodology used for HBV detection.

In a previous study conducted at the same institution where the current study was developed, the authors found 4.4% of OBI in explanted livers from patients with HBsAg-negative cirrhotic who underwent liver transplantation.^26^ In that study, the protocol of Raimondo et al. was employed,^5^ and the investigators analyzed only fresh liver tissue removed from the explanted liver, which provides larger fragments for analysis, facilitating HBV DNA detection.^26^ Conversely, in the present study, we used fragments obtained by percutaneous liver biopsy stored in paraffin blocks. However, contrary to expectations, considering the nature of the material, the frequency of OBI found in the current study was approximately three times higher using the same protocol. It is noteworthy that in the study cited above,^26^ sequencings were performed, and only cases in which the HBV genome was identified were considered OBI cases.

Although nested PCR is considered an efficient molecular tool to detect HBV,^36^ false-positive results may occur when this technique is used to diagnose OBI. It is possible to question whether the presence of cirrhosis makes it difficult to detect HBV DNA. Arguments against this hypothesis are the fact that all patients in the study by Ferrari et al.,^26^ were cirrhotic, and in the current study, only 21.2% showed advanced fibrosis or cirrhosis on histology. Furthermore, in studies by Cacciola et al.,^18^ Sagnelli et al.^37^ and Squadrito et al.,^34^ OBI was also associated with more severe stages of liver fibrosis or cirrhosis. We found no association between the occurrence of OBI and the etiology of the underlying liver disease. The association between OBI and chronic HCV infection has been observed in some investigations.^18,22,37,38^ The small number of OBI cases in our study may explain the lack of this finding in the current study.

The presence of markers of prior HBV infection (anti-HBc and/or anti-HBs) was associated with OBI only when employing the protocol of Raimondo et al.^5^ Previous studies confirm this association,^14,18,39,40^ and some authors suggest that anti-HBc could be considered a sentinel marker of OBI.^14^

The analysis of DNA quality showed that this quality was adequate in only 16.3% of the samples, which may have interfered with the results. The use of paraffinized tissue in molecular biology tests, despite being inferior to the use of fresh material,^41^ allowed for OBI detection in our study.

A difference was found between the two protocols in HBV DNA detection, which reinforces the need for better standardization of the method to diagnose OBI. The protocol by Raimondo et al.^5^ allowed the identification of HBV DNA in more cases when compared with the protocol by Chapel et al.^28^ These authors described a nested-PCR protocol for HBV DNA detection in paraffin-embedded tissue using primers complementary to a conserved region of the S and Pol genes.^28^ However, in the protocol by Raimondo et al.,^5^ primers were used for four conserved regions of the viral genome. It is possible to question whether the large number of primers used in protocol 1 could generate nonspecific binding, resulting in false-positive results. Thus, to increase specificity, we considered actual cases of OBI in which HBV DNA was detected using both protocols.

The low prevalence of OBI in this study limited the comparative analysis of the characteristics of patients with and without OBI. Another limitation of the study was the inability to perform gene sequencing of the positive samples, which occurred due to a technical issue because the tissue samples from several patients were too small. Our results may also be biased when considering the universe of patients with chronic liver disease, as we selected only cases that underwent biopsy.

Of the phases of HBV infection, the least understood phase is OBI.^42^ Several aspects need further investigation, such as the possible influence on the course of associated liver disease, the role of genetic polymorphisms in its development, and the diagnostic value of viral markers. In this context, it was observed that genetic variants of HLA-DP and the presence of anti-HBc may be important predictors of OBI.^43^ On the other hand, Daef et al.^44^ demonstrated that total anti-HBc is an ineffective marker of OBI. The association with HCV infection has been studied by different authors, but the results have been controversial. In some studies, the absence of an interaction between OBI and chronic hepatitis C was observed,^45,46^ while others have identified that some mutations in HBV may favor its occult phenotype in chronic HCV carriers.^47^

OBI has been suggested to be associated with hepatocarcinogenesis. An increasing number of prospective studies and meta-analyses have demonstrated a higher incidence of HCC in patients with HCV infection and OBI, as well as more advanced tumor histological grades and earlier age of HCC presentation compared to patients without OBI. The suggested pathogenic mechanisms of OBI-related HCC include the influence of HBV DNA integration on the hepatocyte cell cycle, production of pro-oncogenic proteins, and persistent low-grade necroinflammation.^48,49^

## CONCLUSION

This study showed a difference in the results of the two protocols, reinforcing the need for better standardization of the method for diagnosing OBI. Additional studies with larger sample sizes are needed to standardize diagnostic methods for OBI. Furthermore, it is important to conduct prospective studies to clarify the actual impact of OBI on the progression of chronic hepatopathies of different etiologies and the role of occult HBV in hepatocarcinogenesis.
